# Anticancer Effects and Mechanisms of Action of Plumbagin: Review of Research Advances

**DOI:** 10.1155/2020/6940953

**Published:** 2020-12-01

**Authors:** Zhenhua Yin, Juanjuan Zhang, Lin Chen, Qingfeng Guo, Baocheng Yang, Wei Zhang, Wenyi Kang

**Affiliations:** ^1^Zhengzhou Key Laboratory of Medicinal Resources Research, Huanghe Science and Technology College, Zhengzhou 450063, China; ^2^Henan Joint International Research Laboratory of Drug Discovery of Small Molecules, Zhengzhou 450063, China; ^3^National R&D Center for Edible Fungus Processing Technology, Henan University, Kaifeng 475004, China

## Abstract

Plumbagin (PLB), a natural naphthoquinone constituent isolated from the roots of the medicinal plant *Plumbago zeylanica* L., exhibited anticancer activity against a variety of cancer cell lines including breast cancer, hepatoma, leukemia, melanoma, prostate cancer, brain tumor, tongue squamous cell carcinoma, esophageal cancer, oral squamous cell carcinoma, lung cancer, kidney adenocarcinoma, cholangiocarcinoma, gastric cancer, lymphocyte carcinoma, osteosarcoma, and canine cancer. PLB played anticancer activity *via* many molecular mechanisms, such as targeting apoptosis, autophagy pathway, cell cycle arrest, antiangiogenesis pathway, anti-invasion, and antimetastasis pathway. Among these signaling pathways, the key regulatory genes regulated by PLB were NF-k*β*, STAT3, and AKT. PLB also acted as a potent inducer of reactive oxygen species (ROS), suppressor of cellular glutathione, and novel proteasome inhibitor, causing DNA double-strand break by oxidative DNA base damage. This review comprehensively summarizes the anticancer activity and mechanism of PLB.

## 1. Introduction

**Figure 1:**
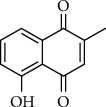
Chemical structure of plumbagin.

Plumbagin (5-hydroxy-2-methyl-1,4-napthoquinone, PLB, Figure 1) is a naturally occurring naphthoquinone isolated from the roots of the medicinal plant *Plumbago zeylanica* L. As a vitamin K3 analog and a prooxidant, PLB has been reported to possess diverse biological activities, such as anti-inflammatory, anticancer, antidiabetic, antioxidant, antibacterial, antifungal, anti-atherosclerosis, and analgesic activities [1–6]. In particular, the anticancer activity and mechanism of PLB have become a research hotspot and increasing attention has been paid by scholars. Some scholars had reviewed the advances in tumor suppression and mechanism by PLB in year 2014 [7] and the cytotoxic potential and pharmaceutical relevance towards cancer therapy [8]. At present, there are few systematic reviews on its anticancer activity.

In this review, we mainly report the recent advances in research on the anticancer activity of PLB and summarized the possible mechanism of PLB, which will provide theoretical references for its clinical application.

## 2. Antitumor Activity

### 2.1. Antibreast Cancer Activity

PLB showed antibreast cancer activity. PLB induced cytotoxicity in human breast cancer cells (MDA-MB-231 and MCF-7) along with cell cycle arrest, DNA damage, apoptosis, and suppression of telomere and telomerase activity [9] and induced cytoplasmic vacuolation and cell cycle G2/M arrest in MDA-MB-231 cells through inhibition of proteasome and disruption of sulfhydryl homeostasis[10]. In endocrine-resistant breast cancer cells (MCF-7/LCC2 and MCF7/LCC9), PLB inhibited their growth, invasion, and metastasis by suppressing *Snail* and altering the expression of other epithelial-mesenchymal transition (EMT) markers [11].

The nuclear factor kappa-B (NF-*κ*B) was usually overexpressed in a subset of HER2-positive breast cancers, and its upregulation was related to the metastatic potential of HER2 overexpressed tumors. Kawiak et al. [12] proved that PLB inhibited the invasion of HER2-overexpressing breast cancer cells BT474 and SKBR3 by inhibiting IKK*α*-mediated NF-*κ*B activation and downregulating NF-*κ*B-regulated MMP-9 expression. In addition, PLB showed different anticancer effects on racially different triple-negative breast cancer cells by the NF-*κ*B pathway and CCL2 release, such as MDA-MB-231 (MM-231) and MDA-MB-468 (MM-468), representing Caucasian Americans and African Americans, respectively [13].

PLB was known to generate ROS in cancer cells [14]. As a ROS inducer, PLB induced apoptosis of defective BRCA1 mutant triple-negative cells and mouse models by ROS-mediated DNA double-strand breaks (DSB) [15, 16] and also induced apoptosis of MCF-7 cells by increasing ROS production and loss of mitochondrial membrane potential. The generation of intracellular ROS could cause the induction of apoptosis *via* the p53-dependent pathway [17, 18].

PLB not only exerted antibreast cancer effect independently but also had synergistic effect with some other drugs, such as reversing the drug resistance of antibreast cancer drugs. One study found that the combination of PLB with zoledronic acid (ZA) could synergistically suppress human breast cancer MDA-MB-231SArfp cells *in vitro* [19], and the mechanism was related to the modulation of Notch-1-Bcl-2 signaling and simulation of the spatial structure of adenosine phosphate, which could competitively inhibit the phosphorylation of c-Jun N-terminal kinase/extracellular signal-regulated kinase (JNK/ERK) in MDA-MB-231[20]. PLB also demonstrated a synergistic inhibitory effect with tamoxifen on the growth of endocrine-resistant breast cancer cells [10], increased paclitaxel-induced cell death, and overcame paclitaxel resistance in MCF-7, BT474, and MDA-MB-468 cells through ERK-mediated apoptosis [21].

According to the above analysis, PLB showed antibreast cancer activity mainly through inhibiting proliferation and invasion as well as promoting apoptosis of breast cancer cells. Meanwhile, the molecular mechanisms were related to the NF-*κ*B, p53-dependent, and JNK/ERK pathways. In addition, the synergistic effect of PLB could reverse the drug resistance of the cancer cells caused by other antibreast cancer drugs.  Table 1 reports a summary of antibreast cancer activity of PLB.

### 2.2. Antihepatoma Activity

PLB had antihepatoma carcinoma cell (HCC) activity. Angiogenesis is one of the hallmarks of cancer, and antiangiogenesis agents or small-molecule inhibitors are effective antitumor agents. PLB might be a promising antiangiogenic drug with significant antitumor activity in HCC. Wei et al. [22] demonstrated the finding that PLB restrained hepatocellular carcinoma angiogenesis (SMMC-7721 and Hep3B) by suppressing the migration and invasion of tumor-derived vascular endothelial cells, which was associated with suppressing the expression of angiogenesis pathways (PI3K-Akt, VEGF/KDR, and angiopoietins/Tie2) and angiogenic factors (VEGF, CTGF, ET-1, and bFGF). In addition, PLB also restrained hepatocellular carcinoma angiogenesis by stromal cell-derived factor (SDF-1)/CXCR4-CXCR7 axis [23].

Previous studies have found that increasing the ROS level inhibited cell proliferation and promoted cell apoptosis, which might be caused by decreasing the cell mitochondrial membrane potential [24, 25]. Zhu et al. further found that PLB could improve the resistance of hepatocellular carcinoma HepG2R cells to sorafenib by increasing the ROS level [26].

PLB inhibited the proliferation of SMMC-7721 cell line in a dose- and time-dependent manner and upregulated the expression levels of autophagy genes and related proteins (LC3, Beclin1, Atg7, and Atg5) that were associated with tumor apoptosis and autophagy in HCC cells and promoted apoptosis and autophagic death [27]. In addition, PLB could induce apoptosis of human HCC SMMC-7721 undergoing epithelial-mesenchymal transition by increasing the caspase-3 protein level and cleaving vimentin [28]. Mammalian target of rapamycin (mTOR) is a serine/threonine protein kinase, and the mTOR signaling pathway has been shown to regulate some cellular processes, including protein synthesis, cell growth, cell proliferation, cell death, and tumor angiogenesis[29, 30]. Therefore, PLB could inhibit proliferation and induce apoptosis of HCC through inhibiting the SIVA/mTOR signaling pathway [31]. According to the above analysis, PLB could inhibit proliferation and induce autophagy and apoptosis of HCC, and the mechanism was likely to be related to prestrain angiogenesis, ROS level, autophagy gene and protein expression levels, caspase-3 protein level, vimentin caspase-3, and SIVA/mTOR signaling pathway.  Table 2 reports a summary of antihepatoma cancer activity of PLB.

### 2.3. Antileukemic Activity

PLB had antileukemic activity. Mammalian thioredoxin reductase (TrxR) isoenzymes were selenocysteine-containing homologous flavin enzymes, which could catalyze NADPH to reduce oxidized thioredoxin and play a key role in regulating a variety of redox signaling pathways [32, 33]. Thus, TrxR might be a drug target for tumor therapy. Zhang et al. [34] demonstrated that PLB selectively targeted TrxR by alkylating the C-terminal redox active site Sec498 of this enzyme to suppress its physiological function, leading to accumulation of ROS, decline of GSH/GSSG ratio, depletion of cellular thiols, and collapse of the intracellular redox homeostasis and eventually promoted oxidative stress-mediated apoptosis of HL-60 cells. In addition, PLB could enhance TRAIL-induced apoptosis in Kasumi-1 cells by ROS-mediated upregulation of DR5 expression, activation of caspase-8, and inhibition of cFLIP expression [35]. PLB was also a potent inhibitor of the NF-*κ*B signaling pathway and suppressor of T-ALL cell proliferation, because PLB induced caspase-dependent apoptosis of MOLT-4 cells and inhibited LPS-induced phosphorylation of p65, and the transcription of NF-*κ*B target genes[36]. PLB effectively inhibited viability and proliferation of chronic lymphocytic leukemia cells (HG3 and MEC-1) through decreasing the ratio of Bcl-2/Bax. In addition, PLB promoted the accumulation of MEC-1 cells in the S phase and blocked cell cycle transition of HG3 cells from G0/G1 to S phase [37]. Based on the above analysis, PLB could inhibit proliferation and induce apoptosis of leukemic cells. The mechanism was likely to be related to targeting thioredoxin reductase, ROS-mediated upregulation of DR5 expression, activation of caspase-8, inhibition of cFLIP expression, decreasing ratio of Bcl-2/Bax, and NF-*κ*B signaling pathway.  Table 3 reports the antileukemic cancer activity of PLB.

### 2.4. Antimelanoma Activity

PLB reduced the proliferation and apoptosis of melanoma tumor cells and had radiosensitizing potential and synergistic inhibitory effect in melanoma tumor cells. For example, PLB caused the reduction of cell proliferation, induction of apoptosis, and disruption of mitochondrial membrane potential in BRAF-mutated (SK-MEL-28, WM35, and RPMI-7951), NRAS-mutated (SK-MEL-119), and BRAF/NRAS wild-type (Hs294T) melanoma cells by ROS-mediated disruption of mitochondrial membrane potential and inhibition of PI3K/AKT/mTOR signaling [38]. Further studies found that PLB also increased the production of ROS and inhibited melanoma cell growth and tumorigenicity by inducing endoplasmic reticulum (ER) stress signaling and DNA damage response (DDR) signaling[39].

The radiosensitizing potential of PLB inhibited the growth of B16F1 melanoma cells *in vitro*, and the mechanism was related to oxidative stress and DNA damage that led to enhanced mitochondria-mediated programmed cell death resulting in cell death. Moreover, it was proved that the ability of PLB to augment ionizing radiation induced tumor cell kill [40].

PLB had synergistic inhibitory effect with other antineoplastic drugs on the growth of melanoma through inhibiting multiple key pathways. The combination of PLB and celecoxib decreased melanoma cell proliferation and retarded vascular development of tumors mediated by inhibition of COX-2 and STAT3 leading to decreased levels of key cyclins key on which melanoma cells were dependent for survival[41].  Table 4 reports the antimelanoma activity.

### 2.5. Antiprostate Cancer Activity

PLB inhibited growth of prostate cancer cells and promoted apoptosis and autophagy. Hafeez et al. [42] confirmed PLB inhibited C57BL/6 wild-type mice tumor growth in intact as well as in a castrated Pten-KO mouse model possibly *via* inhibition of PKCe, Stat3, AKT activation, and epithelial-to-mesenchymal transition (EMT) markers (Vimentin and Slug). PLB also promoted apoptosis and autophagy of prostate cancer cells (PC-3 and DU145) *via* Sirt1 and PI3K/Akt/mTOR-mediated pathways with contribution from AMPK, p38 MAPK, visfatin, and ROS-associated pathways [43] and induced differential proteomic responses in PC-3 and DU145 cells by involving in cell cycle, apoptosis, autophagy, and epithelial-to-mesenchymal transformation pathway [44].

In addition, PLB was proven to suppress BRCA1/2 silenced PCa tumor cells and directly target PCSLCs through the regulation of proliferation and inhibition of stem cell growth [45]. PLB improved the efficacy of androgen deprivation therapy (ADT) in prostate cancer. Abedinpour et al. [46] found PLB was effective in combination with drugs that prevented the synthesis of testosterone or its conversion to dihydrotestosterone (DHT). PLB decreased the androgen receptor (AR) levels *in vitro*, but not with drugs that bind to AR [47].

### 2.6. Antibrain Tumor Activity

Oncogenic transcription factor Forkhead Box M1 (FOXM1) induced the expression of genes involved in cell cycle progression and apoptosis. The activation of FOXM1 signaling was closely related to tumorigenesis [48, 49]. PLB, as a natural FOXM1 downregulator, inhibited proliferation, migration, and invasion of U87 and SHG-44 as well as induced the apoptosis of these cells [50, 51].

The expression of matrix metalloproteinases (MMP), especially MMP-2/-9, accelerated the degradation of ECM leading to the migration and invasion of tumor cells [52]. The PI3K/Akt signal transduction pathway also participated in the migration and invasion of different types of cells [53]. PLB downregulated MMP-2/9 expression and inhibited the PI3K/Akt signaling pathway to inhibit the migration and invasion of U87 and U251 cells [54]. In addition, PLB inhibited the viability and growth of U87 and MG cells and promoted cell cycle arrest at G2/M checkpoint by downregulating the cell cycle marker proteins CDK2 and CDK-4 and increased ROS generation that led to increased mitochondrial depolarization as well as activation of caspase-3/7. Akt/mTOR signaling was the potential target of PLB [55].

PLB induced DNA damage, cell cycle arrest, and apoptosis of brain tumor cells, such as human glioblastoma multiforme cells A172, KNS60, U251MG (KO), and medulloblastoma cells ONS76, and next suppressed the colony-forming ability. These effects were substantiated by the upregulation of E2F1, TNFRSF1A, downregulation of E2F1 genes, along with a drop in MDM2, cyclin B1, survivin, and BCL2 protein expression. PLB induced the elevated levels of the caspase-3/7 activity as well [56]. Some scholars have found that inhibition of telomerase could induce the death of human cancer cells because of the abnormal expression of telomerase in cancer cells, compared with normal somatic cells [57]. This finding was consistent with that of Khaw et al. [56] who proved that PLB inhibited telomerase in brain tumor cells and resulted in telomere shortening following chronic long-term treatment. Additionally, PLB displayed a highly cytotoxic activity on rat glioblastoma C6 cells (IC_50_ = 7.7 ± 0.28 *μ*M) and caused cell death by necrosis, which was related to the increasing amount of intracellular ROS and significantly uncoupled mitochondrial oxidation from phosphorylation impairing ATP production in cells[58].

Previous studies have reported that PLB had the role in inhibiting NADPH oxidase 4 (NOX4) and regulating redox signaling [59] and protected cerebral infarction-reperfusion-induced neurogenic injury in rats through the repression of apoptosis and NF-*κ*B activation [60]. Zhang et al. also found that PLB reduced the ROS production by regulating the expression of NOX4 and downregulated NF-*κ*B signaling, which was induced by oxygen-glucose deprivation/reoxygenation (OGDR). PLB inhibited OGDR inducing the activation of NLRP3 inflammasome. Overall, PLB improved the OGDR-induced neurogenic injury by inhibiting NOX4-derived ROS-activated NLRP3 inflammasome [61].  Table 5 reports a summary of the antibrain tumor activity.

### 2.7. Antitongue Squamous Cell Carcinoma Activity

PLB had antitongue squamous cell carcinoma (TSCC) activity *via* p38 MAPK- and PI3K/Akt/mTOR-mediated and p53/GLUT1/MMP2 pathways.

PLB induced apoptosis and autophagy of SCC25 cells through the p38 MAPK- and PI3K/Akt/mTOR-mediated pathways with the contribution from the GSK3*β* and ROS-mediated pathways [62]. PLB also enhanced proliferation inhibition, apoptosis, and autophagy of PF (cisplatin+5-fluorouracil) on CAL27 cells by inhibiting the PI3K/AKT/mTOR-mediated pathway [63]. In addition, PLB also induced the apoptosis of HSC-3 and SAS cells through caspase-3/7 activation, and the mechanism was related to the generation of ROS, which elicited the loss of mitochondrial membrane potential accompanied with JNK and/or p53 activation [64].

Glucose transporter 1 (GLUT1) played an important role in tumor metastasis [65, 66]. The high expression of GLUT1 was shown in normal TSCC, and PLB inhibited TSCC growth *via* suppressing the PI3K/Akt/GLUT1 pathway [67]. Besides GLUT1, the high expression of matrix metalloproteinase 2 (MMP2) was shown in TSCC, and PLB inhibited the invasion and migration of TSCC *via* the p53/GLUT1/MMP2 pathway [68].

### 2.8. Antiesophageal Cancer

Downregulation of FOXM1 inhibited the growth of esophageal cancer cells [69–70]. This finding was consistent with that of Liu et al. [71] who proved that PLB inhibited the proliferation and induced apoptosis of KYSE-30, KYSE-70, and KYSE-140 ESCC *in vitro* and *in vivo* by the downregulation of FOXM1 expression. In addition, previous studies have demonstrated that the overexpression of PLK1 played an essential role in the proliferation and apoptosis resistance of ESCC cells [72, 73]. Cao et al. [74] found that PLK1 was a critical mediator of PLB-induced AKT inactivation. PLB directly downregulated the PLK1 mRNA expression by abolishing the activity of STAT3, a transcriptional factor of PLK1. Taken together, PLB inhibited the proliferation and survival against KYSE150 and KYSE450 ESCC by abrogating the STAT3-PLK1-AKT signaling.

### 2.9. Antioral Squamous Cell Carcinoma Activity

PLB suppressed the oral squamous cell carcinoma (OSCC) cell growth with IC_50_ values ranging from 3.87 to 14.6 *μ*M[64].

### 2.10. Antilung Cancer Activity

PLB inhibited the proliferation, invasion, and migration of lung cancer cells and induced their apoptosis. Kang et al. found PLB inhibited the rho-associated kinase (ROCK) pathway mediated by the FAK/AKT pathway and suppressed lung metastasis in osteopontin- (OPN-) treated NSCLC A549 cells [75]. PLB inhibited proteasome and disrupted sulfhydryl homeostasis inducing cytoplasmic vacuolation, cell cycle sub G1 arrest, and decreased viability in A549 cells [10].

PLB also inhibited the proliferation and induced the apoptosis of the Lewis lung carcinoma cell line in a dose-dependent manner by downregulating the expression of Bcl-2, VEGF[76], and significantly inhibited the proliferation and invasion of L9981 and NL9980 cells by targeting the IL-6/STAT3 signaling pathway[77, 78].

### 2.11. Antikidney Adenocarcinoma Activity

PLB inhibited proliferation and induced apoptosis of kidney adenocarcinoma 786-O cell, and the cell cycle arrested in S and G2/M phases. The mechanism was related to inducing cell membrane integrity damage, reducing mTOR, BCL2, and MDM2 protein levels, and increasing CDKN1A (p21) transcripts and yH2AX phosphorylation levels [79].

### 2.12. Anticholangiocarcinoma Activity

PLB potently exhibited the inhibitory effect on cholangiocarcinoma (CCA) cell line CL-6 growth (IC_50_ = 24.00 ± 3.33 *μ*M) compared with 5-FU-treated cells (IC_50_ = 1036.00 ± 137.77 *μ*M). PLB induced CL-6 cell apoptosis through the stimulation of caspase 3/7 activities [80].

### 2.13. Antigastric Cancer Activity

PLB was a potential regulator of cellular growth, migration, invasion, and apoptosis *via* the upregulation of SHP1 expression and inhibition of jaK2/Stat3 pathway in gastric cancer cells. PLB also inhibited Stat3 pathway through the induction of SHP1 activity in stomach cancer cells [81].

### 2.14. Anticervical Cancer Activity

PLB induced cytoplasmic vacuolation and cell cycle G2 to M arrest that resulted in the decreasing of cell viability in HeLa cells, which was related to the inhibition of proteasome and disruption of sulfhydryl homeostasis [10].

### 2.15. Antilymphocyte Carcinoma Activity

PLB induced the apoptosis in both mouse and human T-cell lymphoma cell lines *via* increasing the oxidative stress, caspase activity, and loss of mitochondrial membrane potential, and oxidative stress-mediated tumor cytotoxicity operates through sustained JNK activation *via* a novel redox-mediated regulation of MKP-1 and MKP-2[82].

### 2.16. Antiosteosarcoma Activity

PLB had antiosteosarcoma activity, mainly reflected by the fact that PLB could inhibit the proliferation and migration of osteosarcoma and induce apoptosis in a dose-dependent manner. The mechanism was related to the suppression of Ezrin and p-Ezrin, inhibiting VEGF and MMP-2/9 genes and proteins, increasing the intracellular ROS level and activation of the mitochondrial pathway [83–85].

### 2.17. Anticanine Cancer Activity

The Na^+^/K^+^-ATP enzyme (NKA) complex was not only the main regulator of membrane potential but also the target of anticancer therapy. NKA was sensitive to oxidative stress, and the expression of NKA was significantly downregulated in expressing oxidative stress cells, which could easily lead to cell death [86, 87]. PLB, as an oxidative stress-causing agent, showed anticanine cancer activity by regulating the activity of NKA. This conclusion was found by Qi et al. (2017) who proved PLB induced apoptosis of canine cancer cells *via* oxidative stress by inhibition of oxidative phosphorylation. Thus, it was concluded that PLB-induced oxidative stress led to the inhibition of NKA in canine cancer cells [88].

## 3. Conclusions and Future Perspective

PLB, as a vitamin K3 analog and a prooxidant, has been reported to possess anticancer activities on a variety of cancer cells. It showed apoptosis, autophagy, antiproliferative, anti-invasive, antimigration, antiangiogenesis property, and cell cycle arrest against various cancers by targeting multiple signaling pathways, generating excess ROS, and disrupting the function of sulfhydryl homeostasis and proteasomal.

Nowadays, due to the side effects of cancer treatment and drug resistance, cancer treatment is in urgent need of a multitarget treatment. Based on a variety of antitumor mechanisms, and combined with existing radiotherapy and chemotherapy, PLB is expected to become an anticancer drug. However, the therapeutic potential of PLB was hindered because it could not reach the tumor specifically at the therapeutic concentration after intravenous administration, and PLB had no secondary effect on normal tissue. Its use in clinic was further limited by its poor aqueous solubility, spontaneous sublimation property, and rapid elimination *in vivo*[89–91]. But some scholars have found that nano or metal formula could improve this disadvantage, such as PLB-silver nanoparticle [92], yttrium (III) complex of PLB [93], pH-responsive bone-targeting drug delivery system (ZA-anchored bimodal mesoporous silica covered gadolinium(III) upconversion nanoparticles loaded with PLB) [94], and transferrin-bearing liposomes entrapping PLB [91].

The review in this paper will be helpful to the research of PLB as antineoplastic drug and further development of its potential clinical application. Further study is needed to clarify the action mechanism of PLB.

## Figures and Tables

**Table 1:** A summary of antibreast cancer activity.

Models	Biological activities	Action mechanism	Reference
MDA-MB-231	Induce apoptosis, cytoplasmic vacuolation inhibit cell migration	Cell cycle arrest, DNA damage, apoptosis, and suppression of telomere and telomerase activity; inhibition of proteasome and disruption of sulfhydryl homeostasis	[8, 9]
MCF-7	Induce apoptosis and inhibit cell migration	Cell cycle arrest, DNA damage, apoptosis, and suppression of telomere and telomerase activity; increasing of ROS production and MMP loss	[8, 16, 17]
MCF-7/LCC2 and MCF7/LCC9	Inhibit growth, invasion, and metastasis	Suppressing snail and altering the expression of other epithelial-mesenchymal transition (EMT) markers	[10]
BT474 and SKBR3	Inhibit invasion	Inhibiting IKK*α*-mediated NF-*κ*B activation and downregulating NF-*κ*B-regulated MMP-9 expression	[11]
MDA-MB-231	With zoledronic acid synergistically suppress	Modulation of Notch-1-Bcl-2 signaling and simulation of the spatial structure of adenosine phosphate	[18, 19]
MCF-7, BT474, and MDA-MB-468 cells	Increase paclitaxel-induced cell death and overcome paclitaxel resistance	Through ERK-mediated apoptosis	[20]

**Table 2:** A summary of antihepatoma activity.

Models	Biological activities	Action mechanism	Reference
SMMC-7721 and Hep3B	Restrain hepatocellular carcinoma angiogenesis	Suppress the expression of angiogenesis pathways (PI3K-Akt, VEGF/KDR, and angiopoietins/Tie2) and angiogenic factors (VEGF, CTGF, ET-1, and bFGF); by stromal cell-derived factor (SDF-1)/CXCR4-CXCR7 axis	[21, 22]
HepG2R	Improve resistance of hepatocellular carcinoma	Increase the ROS level	[26]
SMMC-7721	Inhibit proliferation, induce apoptosis	Upregulate the expression levels of autophagy genes and related proteins (LC3, Beclin1, Atg7, and Atg5); increase the caspase-3 protein level and cleaving vimentin	[27, 28]
HepG2 and LM3	Inhibit proliferation and induce apoptosis	Inhibit the SIVA/mTOR signaling pathway	[31]

**Table 3:** A summary of antileukemic activity.

Models	Biological activities	Action mechanism	Reference
HL-60	Promote oxidative stress-mediated apoptosis	Targeting thioredoxin reductase	[34]
Kasumi-1	Enhance TRAIL-induced apoptosis	ROS-mediated upregulation of DR5 expression, activation of caspase-8, and inhibition of cFLIP expression	[35]
MOLT-4	Induce apoptosis	Inhibit LPS-induced phosphorylation of p65, and the transcription of NF-*κ*B target genes	[36]
HG3 and MEC-1	Inhibit viability and proliferation	Decrease the ratio of Bcl-2/Bax	[37]

**Table 4:** A summary of antimelanoma activity.

Models	Biological activities	Action mechanism	Reference
SK-MEL-28, WM35, RPMI-7951, SK-MEL-119, and Hs294T	Reduction of cell proliferation, induction of apoptosis, and disruption of mitochondrial membrane potential	ROS-mediated disruption of mitochondrial membrane potential and inhibition of PI3K/AKT/mTOR signaling	[38]
Melanoma cell	Inhibit melanoma cell growth and tumorigenicity	Induce endoplasmic reticulum (ER) stress signaling and DNA damage response (DDR) signaling	[39]
B16F1	Inhibit growth, radiosensitizing potential	Oxidative stress and DNA damage	[40]
Melanoma cell	With celecoxib decrease melanoma cell proliferation and retard vascular development of tumors mediated	Inhibit COX-2 and STAT3	[41]

**Table 5:** A summary of the antibrain tumor activity.

Models	Biological activities	Action mechanism	Reference
U87 and SHG-44	Inhibit proliferation, migration, and invasion and induced apoptosis	Downregulate FOXM1	[50, 51]
U87 and U251	Inhibit migration and invasion	Downregulate MMP-2/9 expression and inhibit PI3K/Akt signaling pathway	[54]
U87 and MG	Inhibit the viability and growth, promoted cell cycle arrest	Downregulate the cell cycle marker proteins CDK2 and CDK-4 and increase ROS generation	[55]
A172, KNS60, U251MG, and ONS76	Induce DNA damage, cell cycle arrest, and apoptosis of brain tumor cells and suppressed the colony-forming ability	Upregulate E2F1 and TNFRSF1A and downregulate E2F1 genes, along with a drop in MDM2, cyclin B1, survivin, and BCL2 protein expression. Induce elevated levels of caspase-3/7 activity as well	[56]
Glioblastoma C6 cells	Display highly cytotoxic activity and cause cell death by necrosis	Increase the amount of intracellular ROS, and significantly uncouple mitochondrial oxidation from phosphorylation impairing ATP production in cells	[58]
Human SH-SY5Y cells	Attenuate oxygen-glucose deprivation/reoxygenation-induced injury	Inhibit NOX4-derived ROS-activated NLRP3 inflammasome	[61]
